# Role of the co-stimulatory molecule inducible T-cell co-stimulator ligand (ICOSL) in the progression of experimental metabolic dysfunction-associated steatohepatitis

**DOI:** 10.3389/fimmu.2023.1290391

**Published:** 2023-11-22

**Authors:** Alessia Provera, Naresh Naik Ramavath, Laila Lavanya Gadipudi, Casimiro Luca Gigliotti, Elena Boggio, Cristina Vecchio, Ian Stoppa, Roberta Rolla, Renzo Boldorini, Mario Pirisi, Carlo Smirne, Emanuele Albano, Umberto Dianzani, Salvatore Sutti

**Affiliations:** ^1^ Department of Health Sciences, Università del Piemonte Orientale, Novara, Italy; ^2^ Department of Pediatrics, Washington University in St. Louis, St Louis, MO, United States; ^3^ Translational Medicine and Interdisciplinary Research Centre for Autoimmune Diseases, Università del Piemonte Orientale, Novara, Italy

**Keywords:** nonalcoholic fatty liver disease, metabolic dysfunction-associated steatotic liver disease, liver fibrosis, chronic inflammation, macrophages, galectin-3, osteopontin

## Abstract

**Background and aims:**

Inducible T-cell Co-Stimulator (ICOS) present on T-lymphocytes and its ligand ICOSL expressed by myeloid cells play multiple roles in regulating T-cell functions. However, recent evidence indicates that reverse signalling involving ICOSL is also important in directing the differentiation of monocyte-derived cells. In this study, we investigated the involvement of ICOS/ICOSL dyad in modulating macrophage functions during the evolution of metabolic dysfunction-associated steatohepatitis (MASH).

**Results:**

In animal models of MASH, ICOS was selectively up-regulated on CD8^+^ T-cells in parallel with an expansion of ICOSL-expressing macrophages. An increase in circulating soluble ICOSL was also evident in patients with MASH as compared to healthy individuals. ICOSL knockout (ICOSL^-/-^) mice receiving choline/methionine deficient (MCD) diet for 6 weeks had milder steatohepatitis than wild type mice. MASH improvement was confirmed in mice fed with cholesterol-enriched Western diet for 24 weeks in which ICOSL deficiency greatly reduced liver fibrosis along with the formation of crown-like macrophage aggregates producing the pro-fibrogenic mediators osteopontin (OPN) and galectin-3 (Gal-3). These effects associated with a selective shewing of F4-80^+^/CD11b^high^ monocyte-derived macrophages (MoMFs) expressing the Triggering Receptor Expressed on Myeloid cells 2 (TREM2) to CD11b^low^/F4-80^+^ cells positive for the Kupffer cell marker C-type lectin-like type 2 receptor (CLEC-2), thus indicating an increased MoMF maturation toward monocyte-derived Kupffer cells.

**Conclusions:**

These results suggest that CD8^+^ T-cells interaction with monocyte-derived macrophages through ICOS/ICOSL critically supports a specific subset of TREM2^+^-expressing cells contributing to the evolution of steatohepatitis. The data also point ICOS/ICOSL dyad as a possible target for therapeutic interventions in MASH.

## Introduction

Nonalcoholic steatohepatitis (NASH) or metabolic dysfunction-associated steatohepatitis (MASH) according to the newly proposed nomenclature (1) represents a common evolution of metabolic dysfunction-associated steatotic liver disease (MASLD), previously known as nonalcoholic fatty liver disease (NAFLD) (1) and a growing cause of end-stage liver disease with a death rate ascribed to MASH-related cirrhosis of about 12-25% (2, 3). Furthermore, MASH evolution to cirrhosis is a rising cause of hepatocellular carcinoma (HCC) in Western countries accounting for 1-38% of the known etiologies of this tumour (4). Current view, points liver inflammation as the driving force for MASH evolution to cirrhosis as well as a specific stimulus for the development of HCC (5). Growing evidence indicates that hepatic inflammation in MASH involves a complex interplay between innate and adaptive immunity (5). In this scenario, innate immune cells and particularly hepatic Ly6c^high^ monocyte-derived macrophages (MoMFs) are seen as the main responsible for producing pro-inflammatory mediators involved in perpetuating hepatocyte injury and liver inflammation. MoMFs also support the activation of hepatic stellate cells (HSCs) and extracellular matrix deposition during NASH progression to fibrosis and cirrhosis (6, 7). Recent high-resolution analytical techniques have evidenced a large phenotypic heterogeneity among MoMFs from human and rodent MASH livers. These phenotypes include “lipid-associated” macrophages (LAM), also known as NASH-associated macrophages (NAMs), “scar-associated” macrophages (SAMs) present in fibrotic niches, and monocyte-derived Kupffer cells (8). Although LAM/NAMs and SAMs share some similarities such as the expression of Triggering Receptor Expressed on Myeloid cells 2 (TREM2) and CD9 (9–12), it is still unclear how these cell subsets relate in supporting inflammation and fibrogenesis.

Lobular inflammation in MASH is also characterized by the liver recruitment and activation of B and T lymphocytes (12, 13). In this setting, the cytokine network generated by Th-1 and Th-17 CD4^+^ T-cells may provide a potent stimulus for the pro-inflammatory activity of macrophages (12). Furthermore, recent studies demonstrate that MASH progression to fibrosis associates with the expansion of CD8^+^ cytotoxic T-lymphocytes. These cells have an “auto-aggressive” behavior toward hepatocytes but can also directly stimulate macrophage and HSC activation (14, 15). What is less clear is how T-cells interact with macrophages in view of emerging data indicating that the progression of chronic liver damage to fibrosis/cirrhosis requires specific cellular interactions in fibrotic cell niches (16). In this latter respect, we observed that, following acute liver injury, CD8^+^ T-cells contribute to liver healing by supporting the survival of reparative TREM-2^+^ macrophages co-expressing the mannose receptor (CD206) and the efferocytosis receptor c-Met Proto-Oncogene Tyrosine Kinase (MerTK) through cellular interactions mediated by the co-stimulatory molecules Inducible T-cell Co-Stimulator (ICOS; CD278) and its ligand ICOSL (CD275, B7h, B7-H2) (17).

ICOS belongs to the CD28 family of co-stimulatory molecules and is mainly expressed by activated T-cells, while ICOSL belongs to the B7 family and is constitutively present on the surface of a variety of cells including macrophages (18). Triggering T-cell ICOS has been shown to modulate several T-cell functions, including cytokine secretion and Follicular T-helper cells (Tfh) differentiation (18). However, recent reports indicate that ICOS/ICOSL interaction can also modulate specific responses in ICOSL-expressing cells through reverse signaling. For instance, ICOSL-mediated signals, beside promoting liver macrophage survival (17), favor dendritic cell maturation (19, 20) and prevent monocyte differentiation to osteoclasts (21).

From this background in the present work, we sought to investigate the possible involvement of the ICOS/ICOSL dyad in modulating macrophage/T-cell interactions in the processes leading to MASH evolution to fibrosis.

## Materials and methods

### Human specimen collection and analysis

Serum samples from 81 Caucasian patients aged ≥18 years (48 males and 33 females) with MASLD or MASH, referring to the Liver Unit of the University Hospital of Novara from 2007 to 2021, were collected at the time of first diagnosis. All patients while undergoing liver biopsy as part of their diagnostic track expressed a valid written informed consent to donate blood samples for scientific analysis. Exclusion criteria were a history of current or past excessive alcohol consumption (>20 g/d for women, >30 g/d for men), coinfection by hepatitis B or hepatitis C or human immunodeficiency virus, autoimmune or genetic liver diseases, current or past HCC or immunosuppressive treatments. Patients were characterized by anthropometric, clinical, and biochemical data and liver biopsies were evaluated for the severity of steatohepatitis and fibrosis according to Kleiner et al. (22). All subjects gave informed consent to the analysis and the study was conducted in strict adherence to the principles of the 1975 Declaration of Helsinki, as revised in 2000. The clinical and biochemical features of patients are reported in **Supplementary Table 1**. Forty age/gender matched healthy subjects were recruited among blood donors. Circulating soluble ICOS and ICOSL were measured in sera by commercial kits supplied by Cloud-Clone Corporation (Wuhan, China).

### Mice and experimental protocol

ICOSL deficient (*ICOSL^-/-^*; B6.129P2-Icosl^tm1Mak^/J) mice in C57BL/6 background were obtained from The Jackson Laboratories (Bar Harbor, Maine, USA) and were breaded along with C57BL/6 wild-type mice in our animal facility. Experimental MASH was induced in eight-week-old male by either feeding with methionine/choline deficient diet (MCD) for 2 or 6 weeks or with a high fat/high carbohydrate diet enriched with 1,25% of cholesterol (Western Diet; WD) for 24 weeks (Laboratorio Dottori Piccioni, Gessate, Italy). Control animals received the same diets supplemented by either choline/methionine or normal chow pellets. In some experiments, WD diet was also administered for 24 weeks to ICOS deficient (*ICOS^-/-^* strain B6.129P2-Icos^tm1Mak^/J; The Jackson Laboratories, Bar Harbor, Maine, USA). All the animals were housed at 22°C in pathogen-free conditions with alternating 12 hours light/dark cycles. The mice were not fasted before sample collections. In all the experiments mice were anaesthetized with isoflurane between 9 a.m. and 12 a.m., and blood was collected by retro-orbital bleeding. Afterwards, the mice were euthanized by cervical dislocation. The experimental procedures complied with the EU guidelines for animal experimentation and were approved by the Italian Ministry of Health.

### Assessment of liver injury

Livers were rapidly removed, rinsed in ice-cold saline, and cut into five pieces. Aliquots were immediately frozen in liquid nitrogen and kept at −80°C until analysis. Two portions of the left lobe from each liver were fixed in 10% formalin for 24h and embedded in paraffin. 4 µM tick liver sections were stained with hematoxylin/eosin using a Roche Ventana HE 600 automatic staining system (Roche Diagnostics International AG, Rotkreuz, Switzerland) and microphotographs were taken using a Nikon Eclips CI microscope fitted with a DSR12 camera (Nikon Europe BV, Amsterdam, Netherlands) using the NIS-Elements F4.60.00 acquisition software. Plasma alanine aminotransferase (ALT) levels and liver triglycerides were determined by spectrometric kits supplied, respectively, by Gesan Production SRL (Campobello di Mazara, Italy) and Sigma Aldrich (Milano, Italy).

### Histology and immunohistochemistry

Formalin-fixed and paraffin-embedded (FFPE) liver sections (4-µm tick) were stained with hematoxylin/eosin using a Roche Ventana HE 600 automatic staining system (Roche Diagnostics International AG, Rotkreuz, Switzerland), while collagen deposition was detected by Picro-Sirius Red staining. Sections were scored blindly for steatosis and lobular inflammation, as described (23). The extension of Sirius Red was quantified by histo-morphometric analysis using the ImageJ software (https://imagej.nih.gov/ij/). Liver macrophages were evidenced on FFPE liver sections by immunofluorescence staining through the combination of biotinylated monoclonal antibody against F4-80 (Miltenyi Biotec S.r.l, Bologna, Italy) and phycoerythrin-conjugated streptavidin (Thermo Fisher Scientific, Milano, Italy) and by immunohistochemistry with the use of goat polyclonal antibodies against galectin-3 and osteopontin provided by, respectively, R&D Systems (Minneapolis, USA) and Abcam (Cambridge, UK) in combination with a horseradish peroxidase polymer kit (Biocare Medical, Concord, CA, USA). Bile ductular cells were stained using anti-SOX9 (Abcam, Cambridge, UK). Microphotographs were taken using a Nikon Eclips CI microscope fitted with 20x0.5 and 40x0.75 PlanFluor lens and a DSR12 camera (Nikon Europe BV, Amsterdam, Netherlands) through the NIS-Elements F4.60.00 acquisition software.

### Flow cytometry analysis of liver leukocytes

Livers were digested by type IV collagenase (Worthington, USA), and intrahepatic leukocytes were isolated by multiple differential centrifugation steps according to Weide et al. (24). The cell preparations were then subjected to red cell lysis by BD FACS™ Lysing Solution (BD Bioscience, San Jose, CA, USA) and stained using combinations of the following monoclonal antibodies: CD45 (Clone 30-F11, Cat. 12-0451-82), CD3 (Clone 17A2, Cat. 17-0032-82) CD4 (Clone GK1.5, Cat. N. 56-0041-80) CD8 (Clone 53-6.7, Cat. 11-0081-82), NK1.1 (Clone PK136, Cat. 12-5941-81), TCRγδ (Clone eBioGL3, Cat. 12-5711-81) Ly6C (Clone HK1.4, Cat. 53-59-32-80), Ly6G (Clone Clone RB6-8C5, Cat. 47-5931-82), CD206 (Clone MR6F3, Cat. 25-2061-80), CD9 (MZ3FCRUO, Cat. 124815), CLEC-2 (17D9/CLEC-2FCRUO, Cat. 146103, TIM-4 (RMT4-54FCRUO, Cat. 130019) eBioscience, (Thermo Fisher Scientific, Milano, Italy), CD11b (Clone M1/70, Cat. 101212), ICOS (Clone 15F9, Cat. 107705), ICOSL (Clone HK5.3, Cat. 107405), F4-80 (Clone BM8, Cat. 123113, Biolegend, San Diego, CA, USA), TREM-2 (Clone 78.18, Cat. MA5-28223, Thermo Fisher Scientific, Milano, Italy). Sample analysis was performed using the Attune NxT flow cytometer (Thermo Fischer Scientific, Waltham, MA, USA) and data were elaborated with FlowJo™ Software (BD Biosciences, San Jose, CA, USA).

### mRNA extraction and real-time PCR

mRNA was extracted from snap-frozen liver fragments using the TRIzol™ Reagent (Thermo Fischer Scientific, Milano, Italy). cDNA was generated from 1 µg of mRNA using the High-Capacity cDNA Reverse Transcription Kit (Applied Biosystems Italia, Monza, Italy) in a Techne TC-312 thermocycler (TecneInc, Burlington NJ, USA). Real-Time PCR was performed in a CFX96™ Real-time PCR System (Bio-Rad, Hercules, California, USA) using TaqMan Gene Expression Master Mix and TaqMan Gene Expression probes for mouse TNF-α (Mm99999068_m1), IL-12p40 (Mm99999067_m1), CD11b (Mm00434455_m1), TREM-1 (Mm01278455_m1), CXCL10 (Mm00445235_m1), ICOSL (Mm00497237_m1), GATA-3 (Mm00484683_m1), TREM-2 (Mm04209422_m1), galectin-3 (Mm00802901_m1), osteopontin (Mm01204014_m1), α1-procollagen (Mm00801666_g1), TGF-β1 (Mm00441724_m1), alpha-SMA (Mm01546133_m1), and beta-actin (Cat. N4352663) (Thermo Fischer Scientific, Milano, Italy). All samples were run in duplicate and the relative gene expression was calculated as 2^-ΔCt^ over that of β-actin gene and expressed as fold increase over the relative control samples.

### Data analysis and statistical calculations

Statistical analyses were performed by SPSS statistical software (SPSS Inc. Chicago IL, USA) using one-way ANOVA test with Tukey’s correction for multiple comparisons or Kruskal-Wallis test for non-parametric values. Significance was taken at the 5% level. Normality distribution was assessed by the Kolmogorov-Smirnov algorithm.

## Results

### MASH associates with changes in the liver distribution of ICOS and ICOSL expressing cells

**Figure 1:**
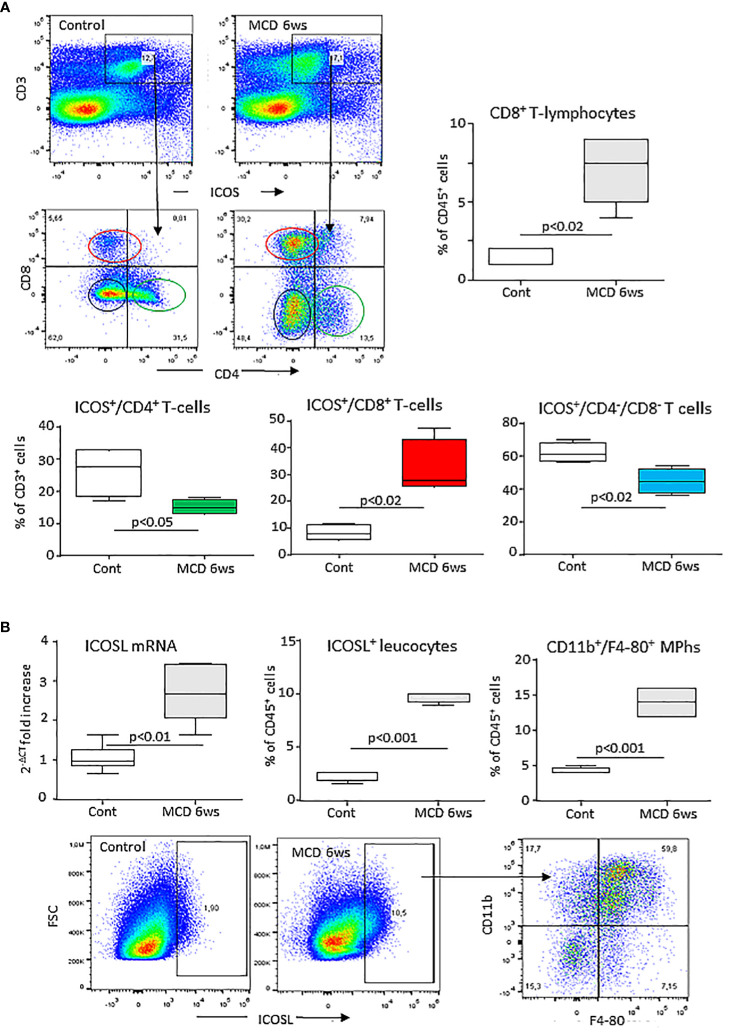
MASH associates with changes in the liver distribution of ICOS and ICOSL expressing cells. Wild type C57BL/6 mice received a methionine/choline deficient (MCD) or control diets for 6 weeks and hepatic lymphocytes and macrophages were analyzed by flow cytometry. **(A)** ICOS expression by the whole pool of CD3^+^ T lymphocytes and localization of ICOS-positive T cell in relation to the distribution of CD4^+^ helper and CD8^+^ cytotoxic and double negative T-cells. **(B)** ICOSL up-regulation within liver myeloid cells and localization of ICOSL-positive cells among CD11b^+^/F4-80 hepatic macrophages. The values refer to 4-5 animals per group and the boxes include the values within 25^th^ and 75^th^ percentile, while the horizontal bars represent the medians. The extremities of the vertical bars (10^th^-90^th^ percentile) comprise 80% percent of the values.

Preliminary analysis of liver T-lymphocytes in mice with experimental MASH induced by 6 weeks feeding with the MCD diet demonstrated a shift in the distribution of ICOS expression among CD3^+^ T-cells. In fact, the development of steatohepatitis was characterized by a 4 folds expansion of ICOS^+^/CD8^+^ T-lymphocytes with a parallel 30-40% lowering of ICOS-expressing CD4^+^ and CD4/CD8-double negative T-cells (**Figure 1A**). These changes partially reflected the modifications in liver T-cell distributions as CD8^+^ T-cell prevalence increased by about 5 folds in mice with MASH (10.2 ± 2.4% vs 1.7 ± 1.2%; p<0.03), without appreciable changes (4.3 ± 1.9% vs 4.1 ± 1.4%) in the fraction of CD4^+^ T cells.

Hepatic inflammation also associated with an up-regulation in the hepatic transcripts for ICOSL which positively correlated with that of the leukocyte activation marker integrin αM (ITGAM or CD11b; r=0.77, p=0.01). Along with that, mice receiving the MCD diet showed an increased prevalence of liver ICOSL-positive myeloid cells (**Figure 1B**) which largely segregated within the expanding fraction CD11b^+^/F4-80^+^ macrophages (**Figure 1B**).

**Figure 2:**
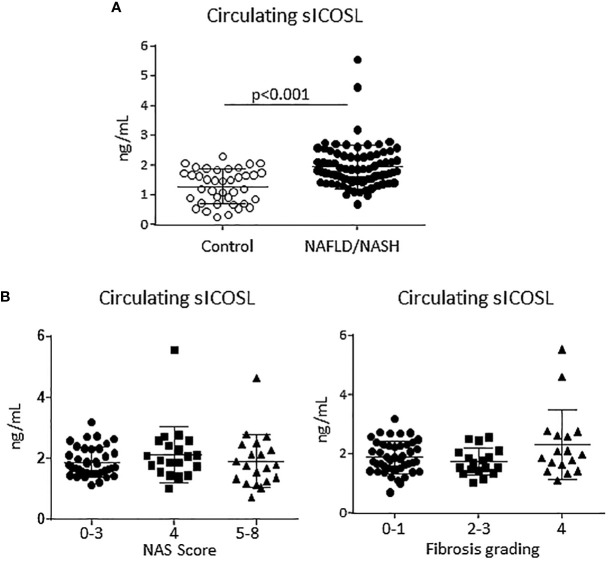
An elevation of the soluble form of ICOSL characterize human MASLD/MASH. **(A)** The soluble forms ICOSL (sICOSL) were measured by ELISA assays in the sera of 81 (48 males and 33 females) patients with and 40 age/gender matched healthy subjects. **(B)** Distribution of sICOSL values according to the severity of liver disease as evaluated by the NAFLD activity score (NAS) and by the grading of hepatic fibrosis among MASLD/MASH patients.

From previous studies showing that ICOS and ICOSL upregulation in human leucocytes correlated with an increase in the circulating levels of their respective soluble forms (25) we evaluated soluble ICOS (sICOS) and ICOSL (sICOSL) in the sera of patients with histologically proven MASLD/MASH. In line with ICOSL upregulation in rodent MASH, we observed that sICOSL was significantly higher in MASLD/MASH patients as compared to healthy individuals (2.03 ± 1.09 ng/mL vs 1.29 ± 0.57 ng/mL; p<0.0001; **Figure 2A**). Among these patients, 36% (29 out of 81 subjects) showed sICOSL levels above the control threshold. However, elevated sICOSL levels were unrelated to the severity of steatohepatitis or the extent of fibrosis (**Figure 2B**), and neither evidenced relation with the presence of metabolic syndrome or diabetes. Conversely, the same patients did not show appreciable changes in sICOS as compared to healthy controls (data not shown).

### ICOSL deficiency improves lobular inflammation

**Figure 3:**
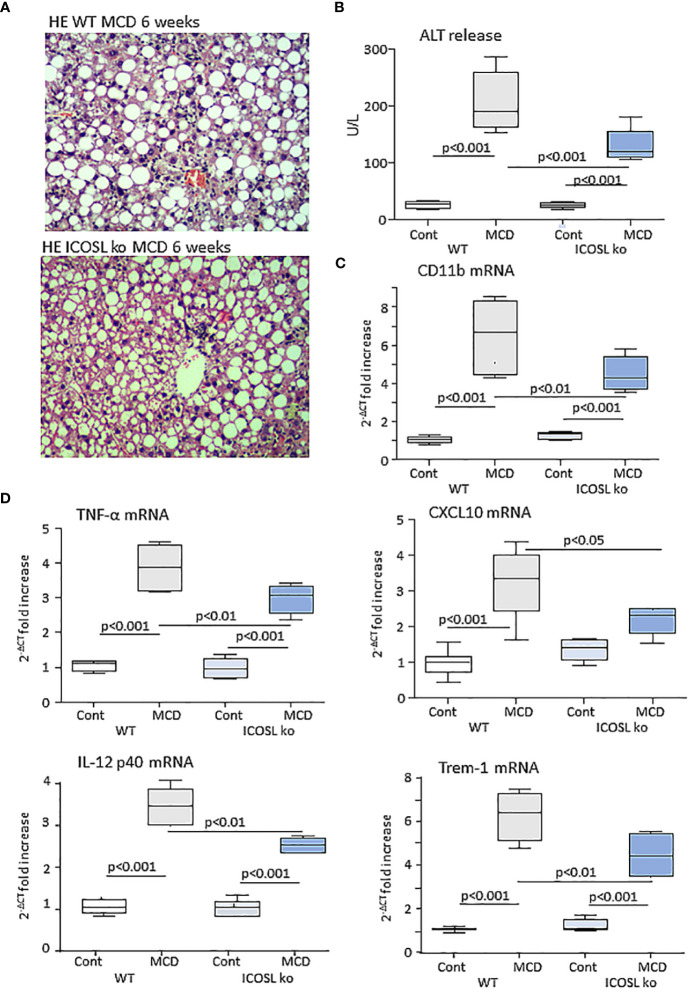
ICOSL ablation improves liver injury and inflammation in mice with MASH. Wild type (WT) and ICOSL deficient (ICOSL ko) mice were fed with either control or methionine choline deficient (MCD) diets for 6 weeks and the severity of steatohepatitis was investigated by: **(A)** Haematoxylin/eosin staining of liver sections (Magnification 200x); **(B)** Alanine aminotransferase (ALT) release; The hepatic mRNA levels of the leucocyte marker CD11b **(C)** and of inflammatory markers TNF-α, CXCL10, IL-12p40 and TREM-1 **(D)**. RT-PCR values are expressed as fold increase of 2^-ΔCT^ over the relative control samples. The values in the panels B, and C refer to 5-7 animals per group and the boxes include the values within 25^th^ and 75^th^ percentile, while the horizontal bars represent the median. The extremities of the vertical bars (10^th^-90^th^ percentile) include 80% of the values.

From these results, we investigated whether the lack of ICOSL might influence inflammatory and immune responses involved in MASH evolution by feeding ICOSL deficient mice (ICOSL^-/-^) with the MCD diet for 6 weeks. Liver histology showed that parenchymal damage and inflammatory infiltrates were decreased in MCD-fed ICOSL^-/-^ mice (2.8 ± 0.43 vs 2.0 ± 0.63; p=0.041) as compared to wild-type littermates without appreciable differences in the extension of steatosis (**Figure 3A**). Transaminase release as well as the transcripts for the leucocyte marker the inflammatory CD11b and markers such as, TNF-α, IL-12p40, CXCL10 and the Triggering Receptor Expressed on Myeloid cells 1 (TREM-1) were also significantly lowered in MCD-fed ICOSL^-/-^ mice than in the corresponding wild-type mice (**Figures 3B–D**).

Although the ICOS/ICOSL dyad is involved in regulating several aspects of T-cell responses (17), the improvement of steatohepatitis observed in ICOSL^-/-^ mice did not involve significant changes in the relative prevalence of liver CD3^+^/CD4^+^ and CD3^+^/CD8^+^ T-lymphocytes (**Supplementary Figure 1A**). Recently, Torres-Hernandez and co-workers (26) reported that, during MASH evolution, ICOS signalling is required for the differentiation of a specific subset of IL-17A producing γδ T-cells involved in modulating hepatic inflammation. In our hands, ICOSL deficiency did not affect γδ T-cell prevalence in mice receiving the MCD diet (**Supplementary Figure 1B**). By contrast, flow cytometry analysis of liver macrophages evidenced a lowering of liver infiltrating F4-80^+^ macrophages which mainly involved the fraction of CD11b^high^/F4-80^+^ MoMFs (**Supplementary Figure 1C**).

### ICOSL influences the progression of experimental MASH to liver fibrosis

**Figure 4:**
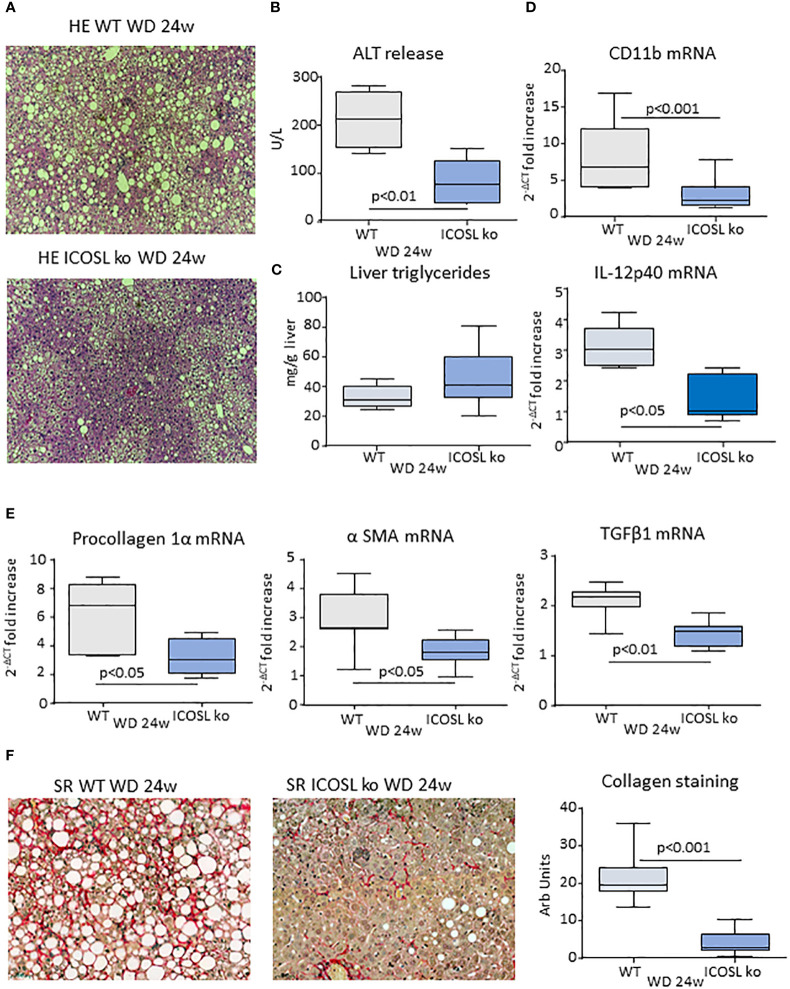
ICOSL modulates progression to liver fibrosis of experimental MASH. Wild type (WT) and ICOSL deficient (ICOSL ko) mice were fed with either control or Western diet (WD) diets for 24 weeks and the severity of steatohepatitis was investigated by: **(A)** Haematoxylin/eosin (HE) staining of liver sections (Magnification 200x); **(B)** Alanine aminotransferase (ALT) release; **(C)** liver triglyceride content; **(D)** hepatic mRNA levels of inflammatory markers CD11b and IL-12p40. The extent of hepatic fibrosis was evaluated by measuring **(E)** fibrosis markers α1-procollagen, α-smooth muscle actin (α-SMA) and Transforming Growth Factor-β1 (TGF-β1) and **(F)** liver collagen deposition as detected by Sirius Red staining (SR). The transcripts for RT-PCR values are expressed as fold increase of 2^-ΔCT^ over the relative control samples. The values in the panels B-F refer to 5-8 animals per group and the boxes include the values within 25^th^ and 75^th^ percentile, while the horizontal bars represent the median. The extremities of the vertical bars (10^th^-90^th^ percentile) include 80% of the values.

Along with the improvement of steatohepatitis ICOSL^−/−^ mice receiving the MCD diet also showed a lowering in the transcripts for fibrosis markers pro-collagen-1α and smooth muscle α-actin (α-SMA) and a reduction in hepatic collagen staining by Sirius Red (**Supplementary Figure 2**), which suggest that ICOSL deficiency might also impact on MASH evolution to fibrosis. To further investigate this issue, we switched to an experimental model of MASH-associated fibrosis more relevant to the human disease, i.e., mice fed with a high fat/high carbohydrate diet enriched with 1.25% of cholesterol (Western Diet; WD) (27). Following 24 weeks on WD diet, morphological and biochemical analysis confirmed that wild type mice developed severe steatohepatitis combined with extensive fibrosis (**Figures 4A–F**). Also in this model, ICOSL deficiency ameliorated the histological scores for lobular inflammation (2.8 ± 0.38 vs 1.7 ± 0.76; p=0.011) as well as transaminase release and pro-inflammatory marker transcripts (**Figures 4A–C**) without affecting hepatic triglyceride accumulation (**Figure 4D**). Moreover, WD-fed ICOSL^−/−^ mice showed an effective prevention of fibrosis as indicated by the lowering in pro-collagen-1α, α-SMA and Transforming Growth Factor β-1 (TGFβ-1) mRNAs (**Figure 4E**) as well as by the morphometric analysis of the diffusion of hepatic collagen fibers in Sirius Red stained liver sections (**Figure 4F**).

### ICOSL deficiency reduces crown-like macrophage aggregates in NASH livers

**Figure 5:**
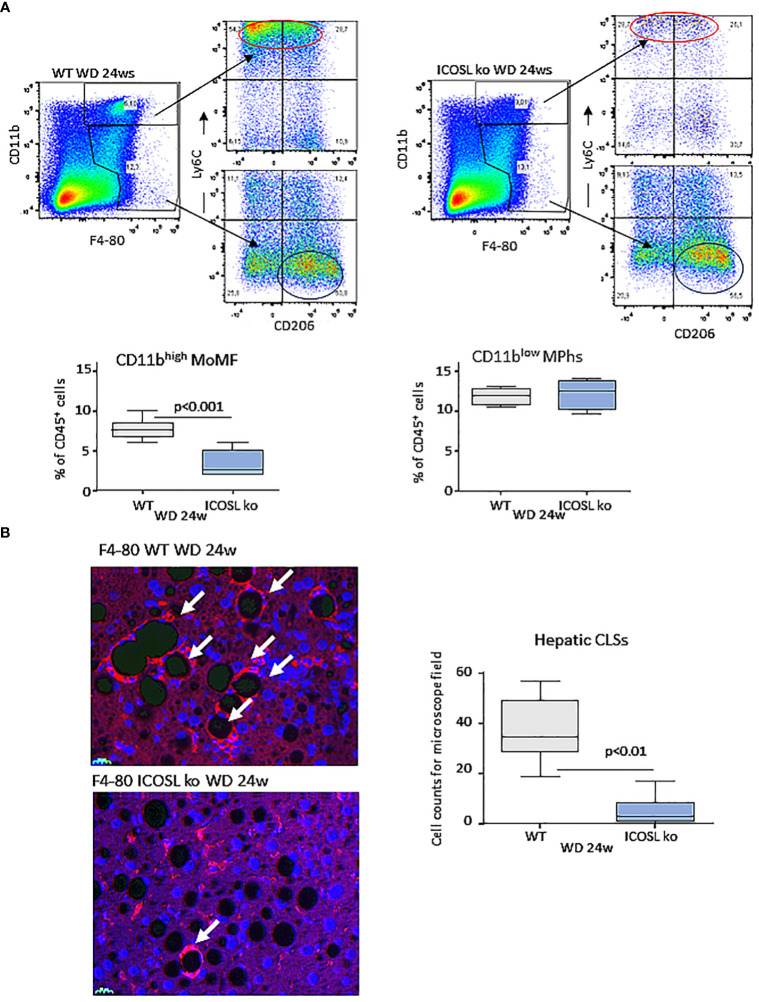
ICOSL deficiency reduced monocyte-derived macrophage distribution and crown-like macrophage aggregates in rodent MASH livers. Wild type (WT) and ICOSL deficient (ICOSL ko) mice were fed with either control or Western (WD) diets for 24 weeks and the animals were investigated for the distribution of hepatic macrophages. **(A)** Flow cytometry analysis of the distribution of Ly6C^high^/CD11b^high^/F4-80^+^ pro-inflammatory monocyte/macrophages (MoMFs) and CD206^+^/CD11b^low^/F4-80^+^ macrophages (MPhs). **(B)** Immunofluorescence (IF) staining of macrophage crown-like structures (hCLSs) using anti-F4-80 antibodies (arrows). The values refer to 4 animals per group ± SD.

As observed in MCD-fed mice, MASH improvement in ICOSL-deficient mice receiving WD was characterized by a significant lowering in the liver fraction of CD11b^high^/F4-80^+^ MoMFs which contains a high proportion of cells expressing the monocyte marker lymphocyte antigen 6 (Ly6C^high^) (**Figure 5A**). Conversely, no appreciable changes were observed in the prevalence of CD11b^low/-^/F4-80^+^/Ly6C^-^ macrophages positive for the mannose binding receptor (MRC1 or CD206) (**Figure 5A**). Beside promoting liver inflammation, MoMFs are responsible for forming aggregates of foamy cells, known as hepatic crown-like structures (hCLS), the frequency of which correlates with the extent of fibrosis (28). Immunofluorescence analysis of liver sections for the macrophage marker F4-80 evidenced an extensive presence of hCLSs throughout the hepatic lobules of WD-fed wild-type mice (**Figure 5B**). In line with the lowering of MoMFs, the absence of ICOSL appreciably decreased hCLS prevalence (**Figure 5B**). These effects did not involve ICOS distribution, as ICOS expressing CD8^+^ T-lymphocytes were comparable in the two strains (15.9 ± 6.1% vs 21.4 ± 7.6% of CD45^+^ cells; p=0.21), suggesting that ICOSL reverse signaling might specifically influence hepatic MoMF behavior.

### ICOSL signaling modulates macrophage phenotype in MASH livers

**Figure 6:**
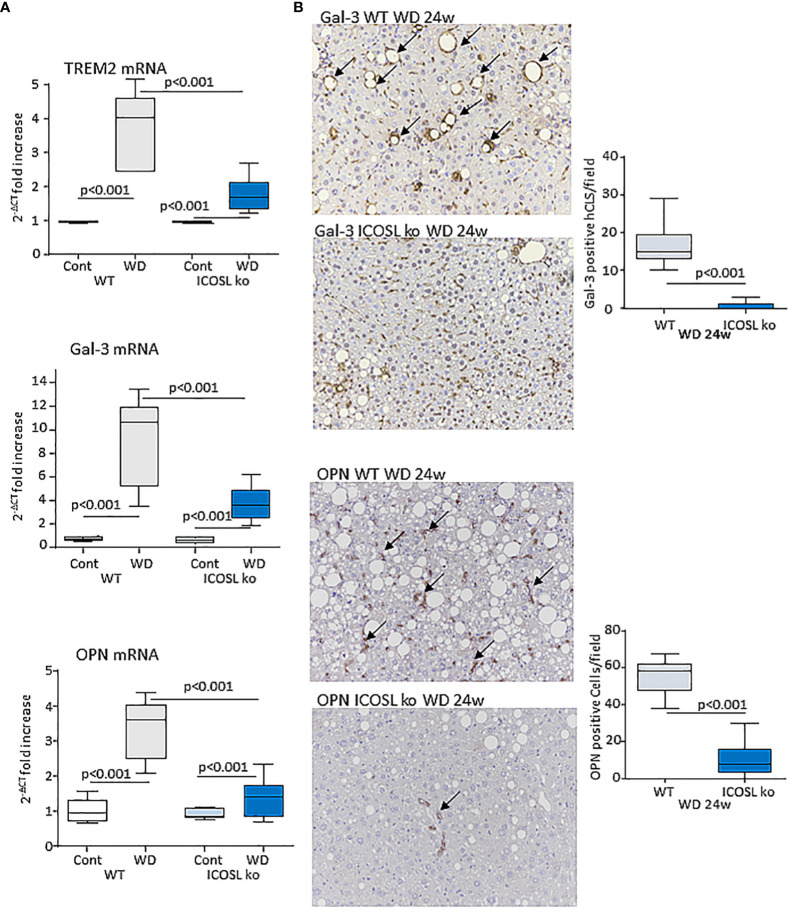
ICOSL signaling modulates macrophage phenotype in rodent MASH livers. Wild type (WT) and ICOSL deficient (ICOSL ko) mice were fed with either control or Western (WD) diets for 24 weeks and investigated for NAM/SAM markers. **(A)** hepatic mRNA levels of TREM2, Galectin-3 (Gal-3) and Osteopontin (OPN). RT-PCR values are expressed as fold increase of 2^-ΔCT^ over the relative control samples. The values in the panel refer to 5-8 animals per group and the boxes include the values within 25^th^ and 75^th^ percentile, while the horizontal bars represent the median. The extremities of the vertical bars (10^th^-90^th^ percentile) include 80% of the values. **(B)** Immunohistochemical detection of Gal-3 and OPN (arrows) in liver sections (magnification 20x). Quantification was performed by counting, respectively, Gal-3 positive hepatic crown-like structures (hCLS) or OPN positive cells in 10 microscopic fields at 20x magnification.

Data from single-cell RNA-sequencing indicates that macrophages forming hCLS express TREM2 (29). These cells, besides having pro-inflammatory activity (30), share with scar-associated macrophages (SAMs) the capacity of producing pro-fibrogenic mediators, such as osteopontin (OPN) and galectin-3 (Gal-3) (29, 31). In line with hCLS expansion liver TREM2, OPN and Gal-3 transcripts were strongly up-regulated in wild-type mice after 24 weeks on the WD (**Figure 6A**). Moreover, immunohistochemistry for Gal-3 and OPN showed a marked positivity for Gal-3 in hCLS, while OPN staining was evident in both hCLSs and biliary tubular cells (**Figure 6B**). The lowering of hCLSs observed in ICOSL^-/-^ mice was accompanied by a reduction in TREM2, OPN and Gal-3 transcripts and Gal-3 staining (**Figure 6B**). Furthermore, OPN-positive macrophages and biliary cells were strongly decreased by ICOSL ablation (**Figure 6B**). The improvement of biliary reaction in ICOSL^-/-^ mice was confirmed by the selective immunostaining of biliary cells with anti-Sox9 antibodies (**Supplementary Figure 3A**).

**Figure 7:**
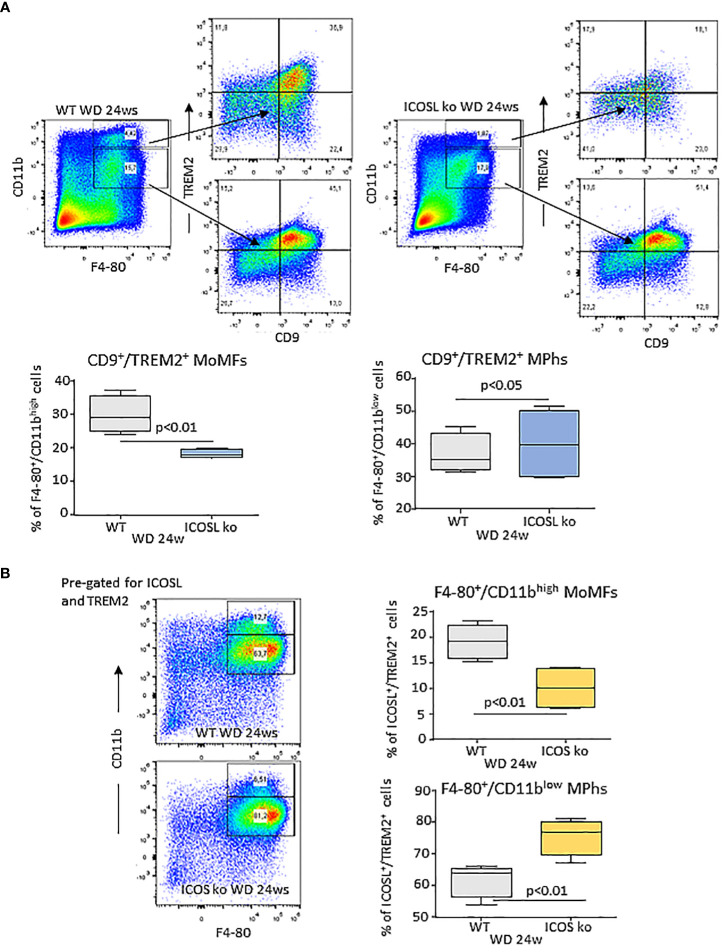
Interference with ICOS/ICOSL dyad selectively affects the distribution of TREM2 expressing cells among MASH-associated hepatic macrophages. **(A)** Distribution of TREM2^+^/CD9^+^ cells among CD11b^high^/F4-80^+^ monocyte/macrophages (MoMFs) and CD11b^low^/F4-80^+^ macrophages (Mphs) in the liver of wild type (WT) and ICOSL deficient (ICOSL ko) fed with either control or Western (WD) diets for 24 weeks. **(B)** Distribution of TREM2^+^/ICOSL^+^ cells among CD11b^high^/F4-80^+^ monocyte/macrophages (MoMFs) and CD11b^low^/F4-80^+^ macrophages (MPhs) in the liver of wild type (WT) and ICOS deficient (ICOS ko) fed with either control or Western (WD) diets for 24 weeks. The values refer to 4 animals per group ± SD.

Recently Hendrikx and coworkers (32) reported that TREM2 upregulation in MASH livers mainly involves MoMFs and monocyte-derived Kupffer cells and that TREM2-expressing macrophages localize within regions characterized by inflammation, cell death, and extracellular matrix remodeling. In our hands, both ICOSL^+^/CD11b^high^ and ICOSL^+^/CD11b^low^ F4-80^+^ cells detected in WD-fed wild type mice express CD9 and TREM2 (**Supplementary Figure 3B**). However, ICOSL deficiency caused a 40% reduction in CD9^+^/TREM2^+^ cells only among CD11b^high^/F4-80^+^ MoMFs (**Figure 7A**). To better follow the fate of TREM2^+^/ICOSL^+^/CD11b^high^/F4-80^+^ MoMFs, we switched to ICOS knockout (ICOS^-/-^) mice that after 24 weeks on the WD also showed a significant reduction in the presence of hCLSs along with less severe steatohepatitis and fibrosis (**Supplementary Figure 4**). In these animals, we observed that the lowering of TREM2^+^/ICOSL^+^/CD11b^high^/F4-80^+^ MoMFs paralleled with the expansion of ICOSL^+^/TREM2^+^ cells within the fraction of CD11b^low^/F4-80^+^ macrophages (**Figure 7B**).

**Figure 8:**
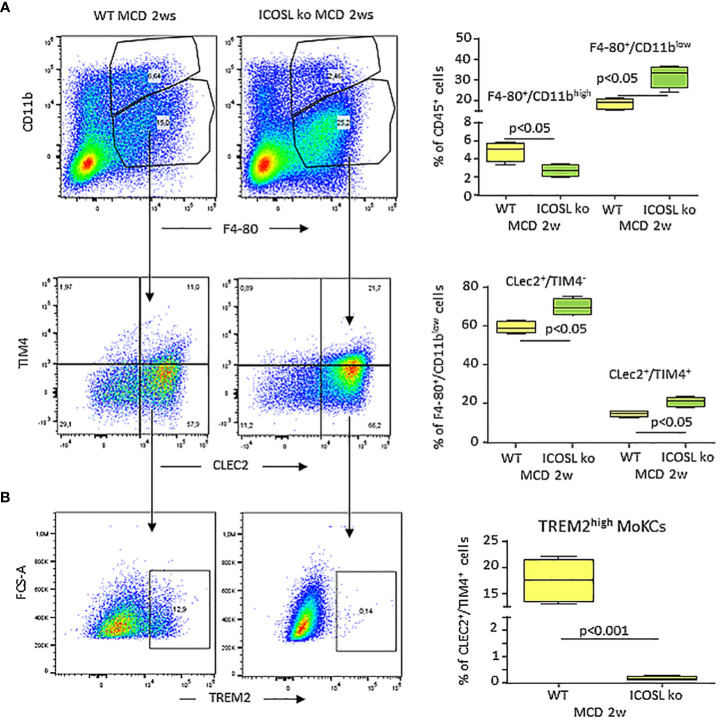
ICOSL modulates monocyte maturation to monocyte derived Kupffer cells (moKCs). Wild type (WT) and ICOSL deficient (ICOSL ko) mice were fed with either control or methionine choline deficient (MCD) for 2 weeks and the animals were investigated by flow cytometry for the distribution of Kupffer cells markers CLEC2 and TIM4 **(A)** and the prevalence of TREM2^high^ cells **(B)** among CD11b^low^/F4-80^+^ macrophages. The values refer to 4 animals per group ± SD.

Interestingly, a 60% loss of CD11b^high^/F4-80^+^ MoMFs and a concomitant expansion of the CD11b^low^/F4-80^+^ macrophages were already evident at the onset of MASH in ICOSL^-/-^ mice receiving the MCD diet for 2 weeks (**Figure 8A**). Also, in these animals MoMF lowering associated with an amelioration in hepatic injury and inflammation (**Supplementary Figure 5A**). We previously reported that reverse ICOSL signaling was required for the survival of reparative TREM-2^+^ macrophages during the healing of acute liver injury (17). In MASH settings, MoMF apoptosis, as evaluated by annexin V staining, slightly increased in MCD-fed ICOSL^-/-^ mice (**Supplementary Figure 5B**), but differences did not reach statistical significance. By analyzing the distribution of the Kupffer cell markers C-type lectin-like type 2 receptor (CLEC-2) and the phosphatidylserine receptor T-cell membrane protein 4 (TIM-4) among CD11b^low^/F4-80^+^ cells we observed that the lack of ICOSL promoted the expansion of both the CLEC-2^+^/TIM-4^-^ and CLEC-2^+^/TIM-4^+^ fractions (**Figure 8B**). Such an effect associated with a lowering of CLEC-2^-^/TIM-4^-^ cells (25.7 ± 3.1% vs 10.8 ± 0.6% of CD11b^low^/F4-80^+^ cells; p=0.014) as well as of TREM2 expression (**Figure 8B**), indicating that ICOSL signalling interfered with MoMF maturation to monocyte-derived Kupffer cells (MoKCs).

All together these data suggested the possibility that reverse ICOSL signaling is critical for supporting a specific subset of TREM2^+^ MoMFs involved in the formation of hCLSs and contributing to the evolution of steatohepatitis.

## Discussion

It is increasingly evident that the pathogenesis of MASLD/MASH involves complex interactions between innate and adaptive immune cells and that such interactions are critical for modulating inflammatory cell behaviour during the disease evolution (16). Here we show that the interaction between CD8^+^ T-lymphocytes and macrophages through the co-stimulatory molecules ICOS and ICOSL is involved in controlling the phenotype of liver infiltrating TREM2^+^ MoMFs sustaining their pro-inflammatory and pro-fibrogenic activities.

Recent studies evidenced that hepatic lipid accumulation promotes the emergence of a macrophage population featuring the expression of TREM2 receptor which can represent more than 60–70% of total liver macrophages (9). These TREM2^+^ cells originate from monocytes and include MoMFs and monocyte derived Kupffer cells (9–11, 29, 32). TREM2 is a transmembrane receptor of the immunoglobulin superfamily that recognizes lipids and apolipoproteins and, by triggering signals via DNAX-activating protein 12 kDa (DAP12), downregulates the transcription of inflammatory genes (33). Consistently, TREM2^+^ macrophages contribute to lipid handling in fatty livers (34), while TREM2 ablation in myeloid cells greatly worsens the severity of rodent MASH (32). However, circulating soluble TREM2 positively correlates with severity of steatohepatitis in patients with MASLD/MASH (32, 35) and the transcriptomic profiles of hepatic macrophages in TREM2^+^ spots reveal an enrichment of genes related to inflammation and extracellular matrix remodeling (32). These contrasting observations and the fact that TREM2 expression characterizes fibrosis-associated SAMs (11) and macrophages forming hepatic crown-like structures (hCLSs) (29), likely reflect the heterogeneity of TREM2^+^ liver macrophages in MASH.

We have observed that ICOSL is upregulated in MASH livers, being expressed by both TREM2^+^/CD9^+^/CD11b^high^ MoMFs and TREM2^+^/CD9^+^/CD11b^low^ macrophages. However, interfering with reverse ICOSL signaling selectively affects the former ones. The lack of ICOSL not only reduces CD11b^high^/Ly6C^high^/TREM2^+^ MoMFs but also prevents the formation of hCLS producing pro-fibrogenic mediators, such as osteopontin (OPN) and galectin-3 (Gal-3). These effects are associated with improvement of steatohepatitis and marked reduction in MASH progression to fibrosis, indicating that ICOSL signaling is critical for supporting a specific subset of TREM2^+^ MoMFs involved in the formation of hCLSs and contributing to the evolution of steatohepatitis. These cells have several features in common with NAMs/SAMs cells previously described in human and rodent MASH livers (9–11) and might represent the fraction of TREM2 macrophages detected in areas of inflammation and extracellular matrix remodeling (32) possibly involved in sustaining the evolution of steatohepatitis.

**Figure 9:**
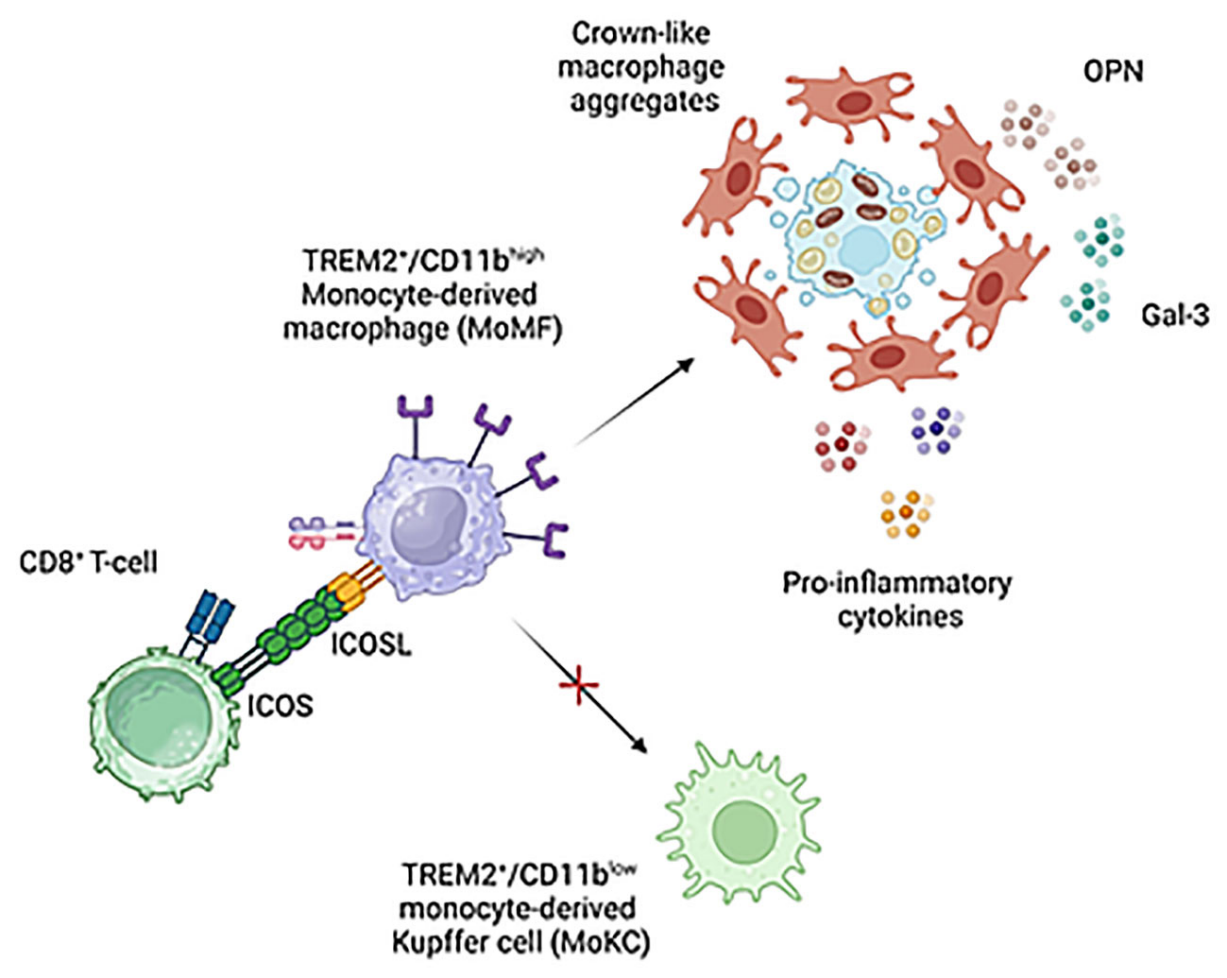
Proposed mechanisms for the modulation of liver macrophages phenotype by ICOS/ICOSL interaction. ICOS expressing CD8^+^ T-cells can contribute to support pro-inflammatory and pro-fibrogenic TREM2^+^/ICOSL^+^ MoMFs involved in forming crown-like aggregates by preventing their differentiation to monocyte-derived Kupffer cells. Image created with BioRender.com.

We have previously reported that ICOSL-mediated signals have anti-apoptotic effects in reparative TREM2^+^ macrophages during the healing of acute hepatic injury in mice poisoned with carbon tetrachloride (CCl_4_) (17). Although we cannot exclude a pro-survival action of ICOSL in MASH associated MoMFs, the main ICOSL function relies in preventing acquisition by TREM2^+^ MoMFs of Kupffer cell like phenotype characterized by the lowering of both Ly6C and CD11b expression and the upregulation of CLEC-2 lectin (**Figure 9**). It is now well established that Kupffer cells are depleted during MASH due to an impaired self-renewal and that inflammatory MoMFs replete the vacant niches acquiring a mixed phenotype of monocyte derived Kupffer cells (MoKCs) (36). Despite MoKCs are still partly immature and display more inflammatory traits than embryonically derived Kupffer cells, during differentiation to Kupffer cells MoMFs lose most of pro-inflammatory and pro-fibrogenic features (31). On the same vein, we have observed that MASH regression in mice treated with the anti-inflammatory protein Annexin A1 is characterized by a marked decrease of hCLSs and the increased in prevalence of CLEC-2^+^/TIM-4^+^ MoKCs (37). Thus, the capacity of ICOSL-derived signals of interfering with MoMF maturation to MoKCs emerges as an important factor in contributing to MASH progression, explaining the improvement of steatohepatitis and fibrosis observed in ICOSL-deficient mice. Interestingly, an increase in circulating soluble ICOSL is also evident among MASLD/MASH patients although without relation with the severity of steatohepatitis or the extent of fibrosis.

At present, it is not known how ICOSL-mediated signals can interfere with the niche stimuli and the combination of transcription factors driving Kupffer cell differentiation (10). Nonetheless, our data suggest that the presence of ICOS-expressing liver infiltrating CD8^+^ T-cells might represent a key factor in modulating MoMF behavior in MASH hepatic milieu. The expansion of activated cytotoxic T-cells is a feature of MASH in both rodents and humans and their ablation is effective in ameliorating steatohepatitis in wild-type mice receiving a high fat/high carbohydrate diet (12, 13). Nonetheless, the mechanisms implicating CD8^+^ T cells in the pathogenesis of MASH are not well characterized. Wolf and coworkers have shown that β2m^-/-^ mice lacking CD8^+^ T- and NKT cells are protected from steatohepatitis in relation to the capacity of CD8^+^ T- and NKT cells to produce LIGHT (38). More recently, Dudek and co-workers (10) have reported that metabolic stimuli induce a specific CD8^+^ T-cell subset combining CXCR6 liver residency marker, effector features and the expression of programmed cell death protein 1 (PD1) responsible for antigen independent hepatocyte killing (10). However, IL-10-expressing CD8^+^ T cells isolated from the livers of mice with obesity-associated NASH have also the capabilities of promoting hepatic fibrosis by directly stimulating macrophage and HSC activation (15). Along this line, we have observed that MASH progression is characterized by the selective expression of ICOS among CD8^+^ T-cells which parallels with the up-regulation of ICOSL by liver infiltrating macrophages. Moreover, ICOS ablation improves MASH by reducing TREM2^+^ MoMFs and the formation of hCLSs. On these bases, we propose that ICOS expressing CD8^+^ T-cells can contribute to MASH pathogenesis by supporting pro-inflammatory and pro-fibrogenic TREM2^+^/ICOSL^+^ MoMFs (**Figure 9**). However, considering that, beside macrophages, ICOSL is also expressed by several other cell types including fibroblasts, we can not exclude that ICOS/ICOSL interaction might contribute to MASH evolution through additional mechanisms.

To our knowledge the only other study that has so far addressed the role of ICOS/ICOSL in the progression of chronic tissue injury is a work by Tanaka and coworkers (39) who have reported that ICOS deficiency prevents bleomycin induced lung fibrosis in mice. However, in Tanaka’s work the severity of lung fibrosis inversely correlated with ICOSL expression and ICOSL deficiency aggravated it (39). The partial inconsistency of these results with our data might be explained by the difference in the experimental systems used as well as by recent evidence indicating that ICOSL has other ligands since by interacting with α_v_β_3_ integrin it mediates kidney protective actions (40) while through osteopontin binding it regulates angiogenic effects in tumors (41).

In conclusion our data demonstrate the implication of ICOS/ICOSL dyad in modulating the evolution of MASH and suggest that the interaction between ICOS^+^/CD8^+^ T lymphocytes and TREM2^+^ MoMFs through reverse ICOSL signalling might represent a novel mechanism by which adaptive immunity contributes to sustain hepatic inflammation and fibrosis in MASH. These results might have possible clinical implications in development of new approaches for the treatment of MASH since monoclonal antibodies targeting ICOS and ICOSL are already under trial for immune-modulatory therapies in cancer.

## Data availability statement

The raw data supporting the conclusions of this article will be made available by the authors, without undue reservation.

## Ethics statement

Ethical approval was not required for the studies involving humans because all patients while undergoing liver biopsy as part of their diagnostic track expressed a valid written informed consent to donate blood samples for scientific analysis. The studies were conducted in accordance with the local legislation and institutional requirements. The human samples used in this study were acquired from gifted from another research group. Written informed consent to participate in this study was not required from the participants or the participants’ legal guardians/next of kin in accordance with the national legislation and the institutional requirements. The animal study was approved by Italian Ministry of Health Direzione Generale della Sanità Animale e dei Farmaci Veterinari Viale Giorgio Ribotta 5 - 00144 ROMA. The study was conducted in accordance with the local legislation and institutional requirements.

## Author contributions

AP: Writing – review & editing, Formal Analysis, Investigation, Methodology. NR: Investigation, Methodology, Writing – review & editing. LG: Investigation, Methodology, Writing – review & editing. CG: Investigation, Writing – review & editing, Data curation. EB: Data curation, Investigation, Writing – review & editing. CV: Investigation, Writing – review & editing. IS: Investigation, Writing – review & editing. RR: Investigation, Writing – review & editing. RB: Writing – review & editing, Formal Analysis. MP: Writing – review & editing, Supervision. CS: Writing – review & editing, Data curation. EA: Writing – review & editing, Writing – original draft. UD: Funding acquisition, Supervision, Writing – review & editing. SS: Methodology, Project administration, Validation, Writing – original draft, Writing – review & editing.

## Supplementary material

Supplementary Table 1 Demographic and Clinical Features of MASDL/MASH patients involved in the study.

Supplementary Figure 1 Effects of ICOSL ablation on the liver distribution of T-lymphocytes and macrophages.

Supplementary Figure 2 ICOSL depletion improves liver fibrosis in mice with experimental MASH.

Supplementary Figure 3 Immunohistochemical staining of liver sections for the biliary cell markers Sox9 and distribution of ICOSL+ cells among TREM2+/CD9+ hepatic macrophages.

Supplementary Figure 4 Effects of ICOS deficiency on liver macrophages and MASH progression to liver fibrosis.

Supplementary Figure 5 Effects of ICOSL ablation on the onset of experimental MASH in mice.
