# Supplementary material for: Role of the co-stimulatory molecule inducible T-cell co-stimulator ligand (ICOSL) in the progression of experimental metabolic dysfunction-associated steatohepatitis
Source: Front Immunol. 2023 Nov 22;14:1290391. doi: 10.3389/fimmu.2023.1290391 (PMC10702974; doi:10.3389/fimmu.2023.1290391)
Supplement: Supplementary Table 1 — Demographic and Clinical Features of MASDL/MASH patients involved in the study. [file DataSheet_1.pdf]

Role of the co-stimulatory molecule inducible T-cell co-stimulator ligand (ICOSL) in the progression of experimental metabolic dysfunction-associated steatohepatitis.

Supplementary material

Supplementary Table 1. Demographic and Clinical Features of MASLD/MASH patients involved in the study.

Patient Characteristic	Value
Male sex, n (%)	48 (59%)
Age, years	55.0 (44.2-60.5)
ALT, U/L	46 (29-72)
GGT, U/L	46 (30-99)
Total bilirubin, mg/dL	0.7 (0.6-1.0)
International Normalized Ratio, Units	1.0 (1.0-1.1)
Platelets, x10^9/L	192 (164-251)
Creatinine, mg/dL	0.8 (0.7-0.9)
Albumin, g/L	44 (40-46)
Liver histology*	
NAS components	
Steatosis, n (%) for 0, 1, 2, 3	0 (0%), 29 (36%), 35 (43%), 17 (21%)
Lobular inflammation, n (%) for 0, 1, 2, 3	16 (20%), 51 (63%), 13 (16%), 1 (1%)
Ballooning, n (%) for 0, 1, 2	41 (50%), 33 (41%), 7 (9%)
NAS**, total score	4 (2-4)
NAS, n (%) for < 4, 4, ≥ 5	40 (49%), 21 (26%), 20 (25%)
Fibrosis staging, n (%) for 0, 1, 2, 3, 4	26 (32%), 20 (25%), 7 (9%), 11 (13%), 17 (21%)
MELD score***	7 (6-8)
Cardiometabolic criteria	
Fasting glucose, mg/dL	105 (96-126)
Fasting insulin, mIU/mL	15.9 (11.7-28.4)
Body mass index, kg/m^2	28.7 (26.1-33.2)
Body mass index ≥ 30 kg/m^2, n (%)	36 (44%)
Impaired glucose metabolism, n (%) for IGT, diabetes mellitus, any defect	21 (26%), 7 (9%), 23 (28%), 51 (63%)
Total cholesterol, mg/dL	177 (148-200)
HDL cholesterol, mg/dL	45 (38-52)
LDL cholesterol, mg/dL	98 (80-120)
Triglycerides, mg/dL	129 (92-158)
Hypertension, n (%)	50 (62%)
Metabolic syndrome****, n	51 (63%)

The data are medians (range) for continuous variables, and as frequencies (%) for categorical variables. IFG: impaired fasting glucose; IGT: impaired glucose tolerance; MELD: Model for end-stage liver disease; NAS: NAFLD activity score; ALT: alanine aminotransferase; GGT: gamma-glutamyl transferase; HDL: high-density lipoprotein; LDL: low-density lipoprotein; NCEP: National Cholesterol Education Program; ATP III: Adult Treatment Panel III. *According to Kleiner et al. classification; **obtained by the sum of scores for steatosis, lobular inflammation and ballooning; ***for cirrhotic patients; ****according to NCEP ATPIII classification.

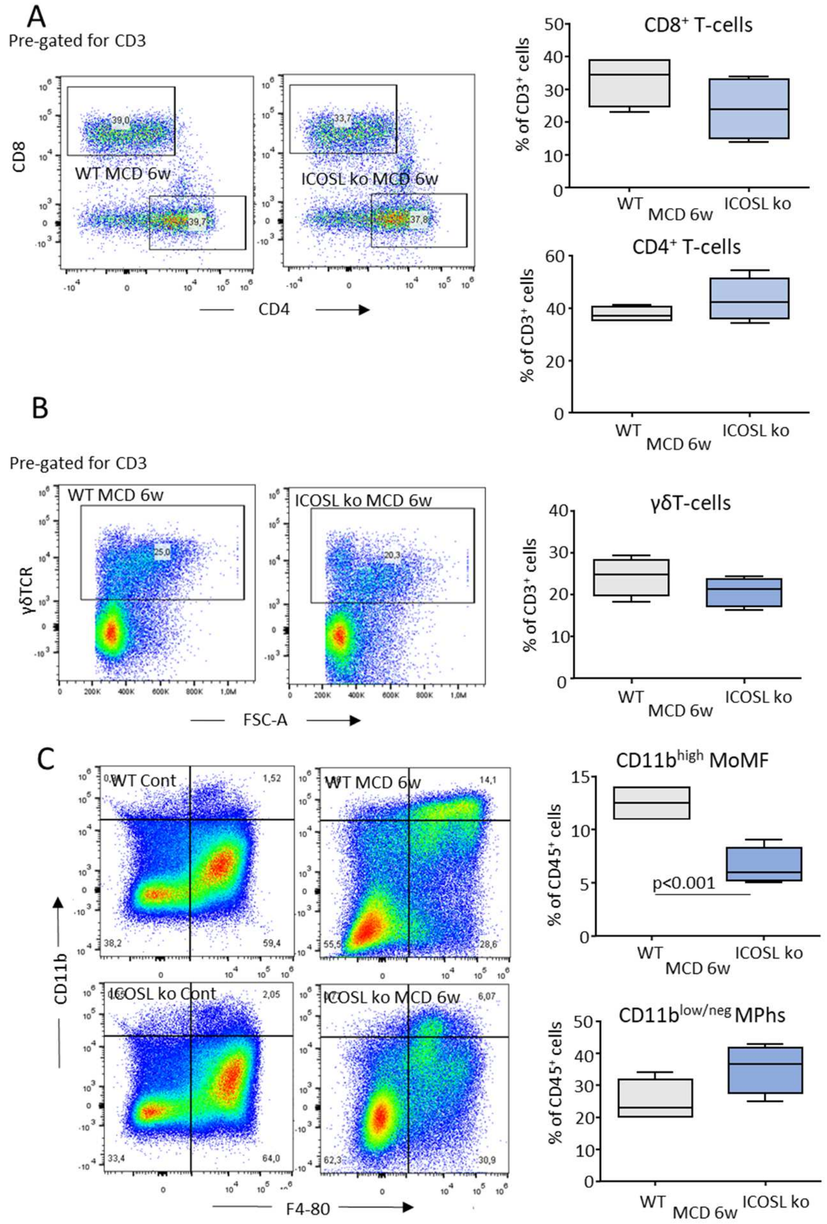

Supplementary Figure 1: Effects of ICOSL ablation on the liver distribution of T-lymphocytes and macrophages.

Wild type C57BL/6 and ICOSL deficient (ICOSL ko) mice received a methionine/choline deficient (MCD) or control diets for 6 weeks and hepatic lymphocytes and macrophages were analyzed by flow cytometry. (Panel A) Prevalence of hepatic $CD4^+$ and $CD8^+$ T-cells. (Panel B) Liver distribution of γδ T-cell among $CD3^+$ T lymphocytes. (Panel C) Changes of $CD11b^{high}/F4\text{-}80^+$ monocyte-derived macrophages (MoMFs) and $CD11b^{low/-}/F4\text{-}80^+$ macrophages (MPhs). The values refer to 4 animals per group and the boxes include the values within 25th and 75th percentile, while the horizontal bars represent the medians. The extremities of the vertical bars (10th-90th percentile) comprise 80% percent of the values.

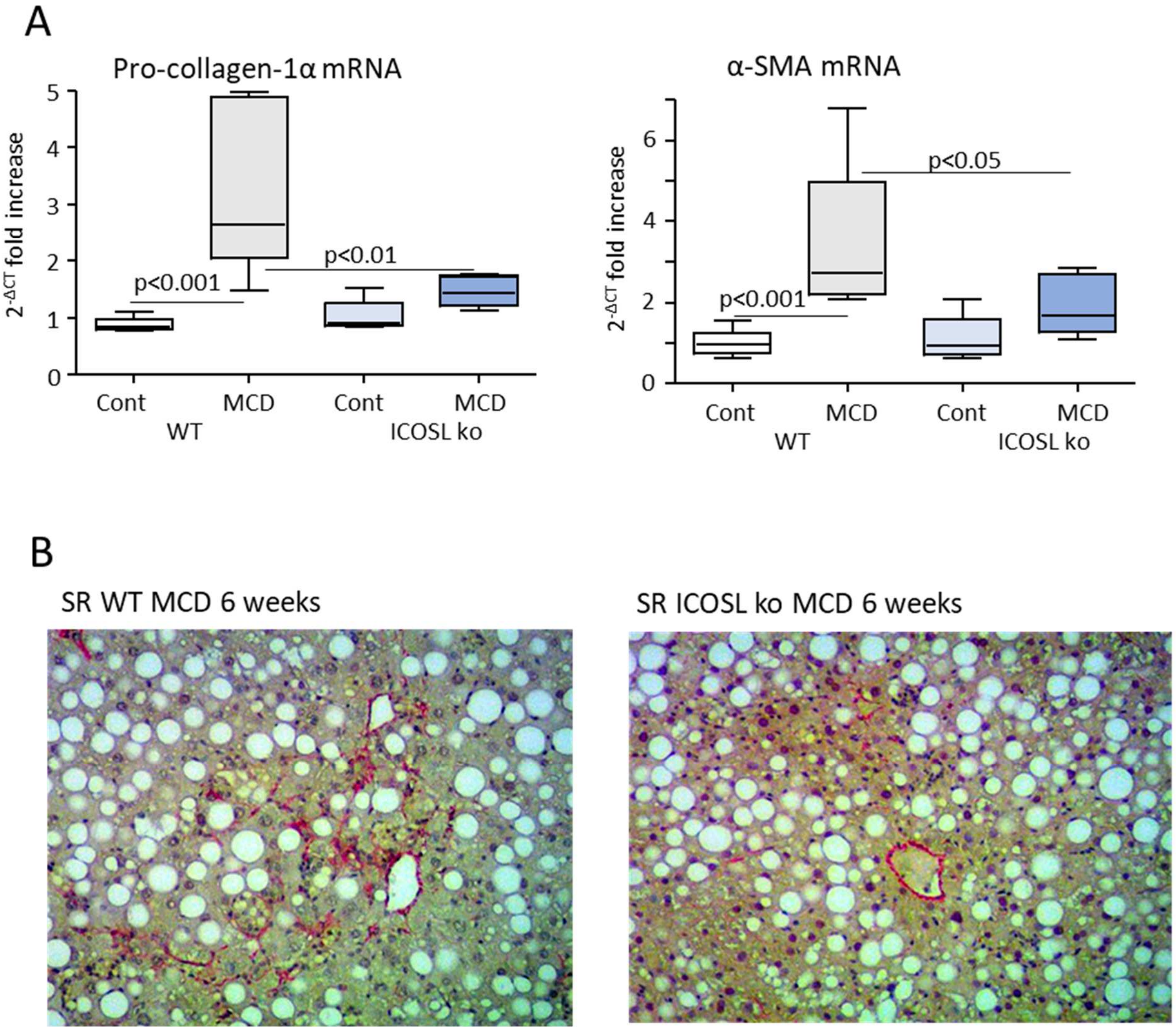

Supplementary Figure 2: Depletion of ICOSL improves liver fibrosis in mice with experimental MASH.

Wild type (WT) and ICOSL deficient (ICOSL ko) mice were fed with either control or methionine choline deficient (MCD) diets for 6 weeks and the MASH evolution to fibrosis was investigated by mRNA expression of fibrosis markers α1-procollagen and α-smooth muscle actin (α-SMA) (Panel A) and by liver collagen staining with Sirius Red (SR) (Panel B; magnification 200x). The transcripts for RT-PCR values are expressed as fold increase of $2^{-\Delta CT}$ over the relative control samples.

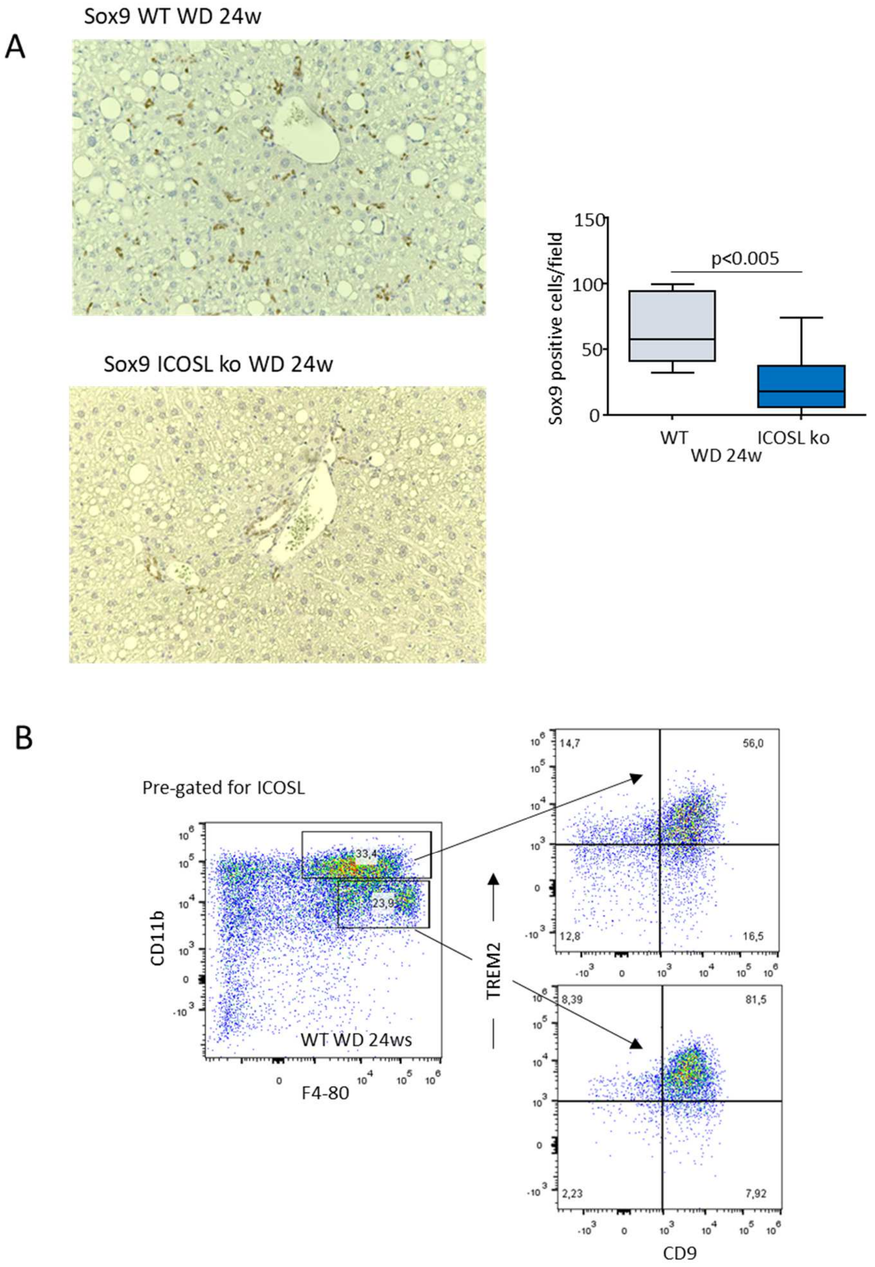

Supplementary Figure 3:

(Panel A) Immunohistochemical staining for the biliary cell markers Sox9 of liver sections from wild type (WT) and ICOSL deficient (ICOSL ko) mice were fed with either control or Western (WD) diets for 24 weeks. Quantification was performed by counting, Sox9 positive cells in 10 microscopic fields at 20x magnification. (Panel B) Distribution of $ICOSL^{+}$ cells among $TREM2^{+}/CD9^{+}$ hepatic macrophages in wild-type mice receiving WD.

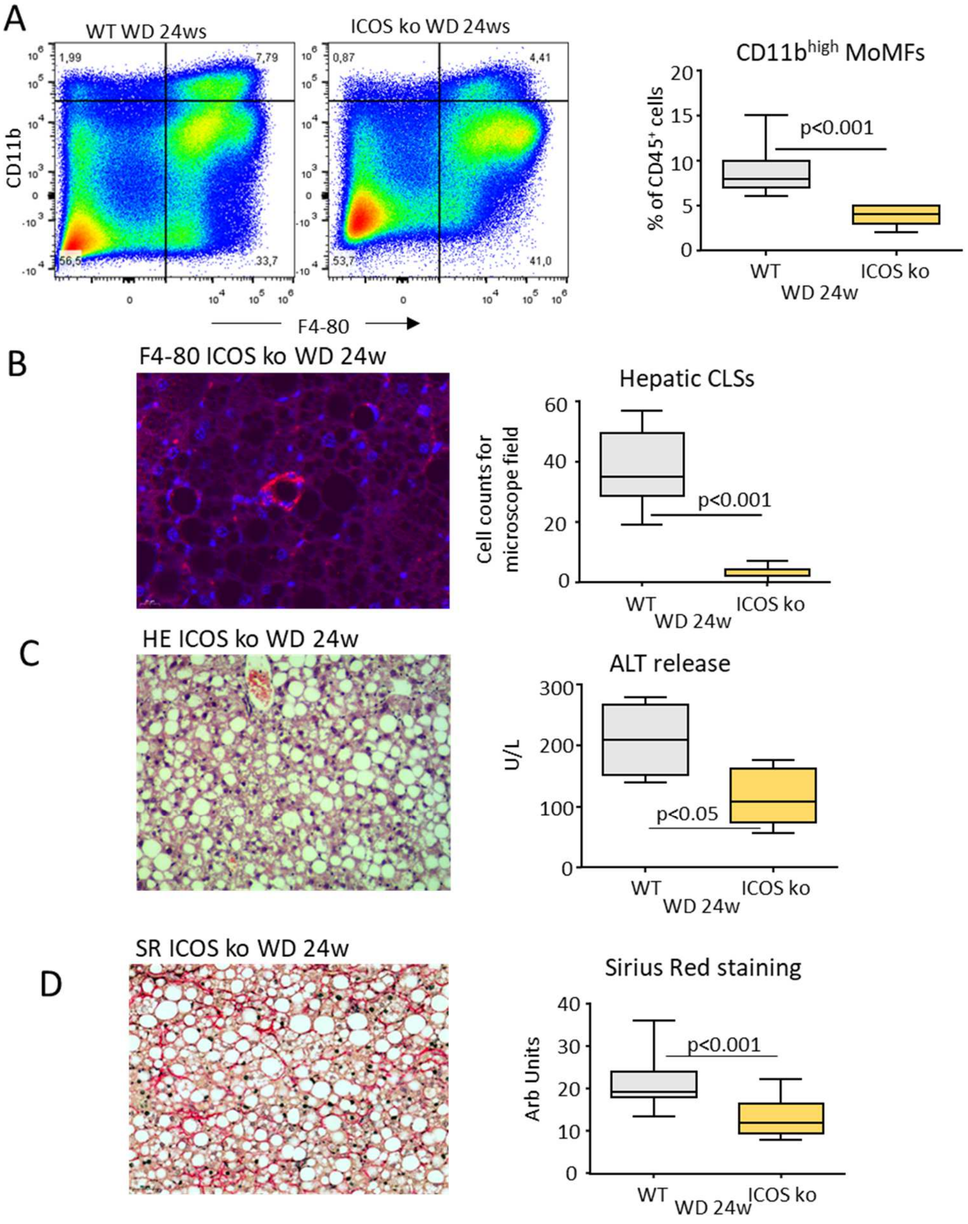

Supplementary Figure 4: Effects of ICOS deficiency on liver macrophages and MASH progression to liver fibrosis.

Wild type (WT) and ICOS deficient (ICOS ko) mice were fed with either control or Western diet (WD) diets for 24 weeks and the effects on steatohepatitis were investigated by: (Panel A) Flow cytometry analysis of the distribution of CD11b^{high}/F4-80^{+} monocyte/macrophages (MoMFs). (Panel B) Immunofluorescence (IF) staining of macrophage crown-like structures (hCLSs) using fluorescent anti-F4-80 antibodies. (Panel C) Haematoxylin/eosin (HE) staining of liver sections (Magnification 200x) and alanine aminotransferase (ALT) release; (Panel D) Liver collagen deposition as detected by Sirius Red staining (SR). The values in the panels refer to 4-6 animals per group and the boxes include the values within 25th and 75th percentile, while the horizontal bars represent the median. The extremities of the vertical bars (10th-90th percentile) include 80% of the values.

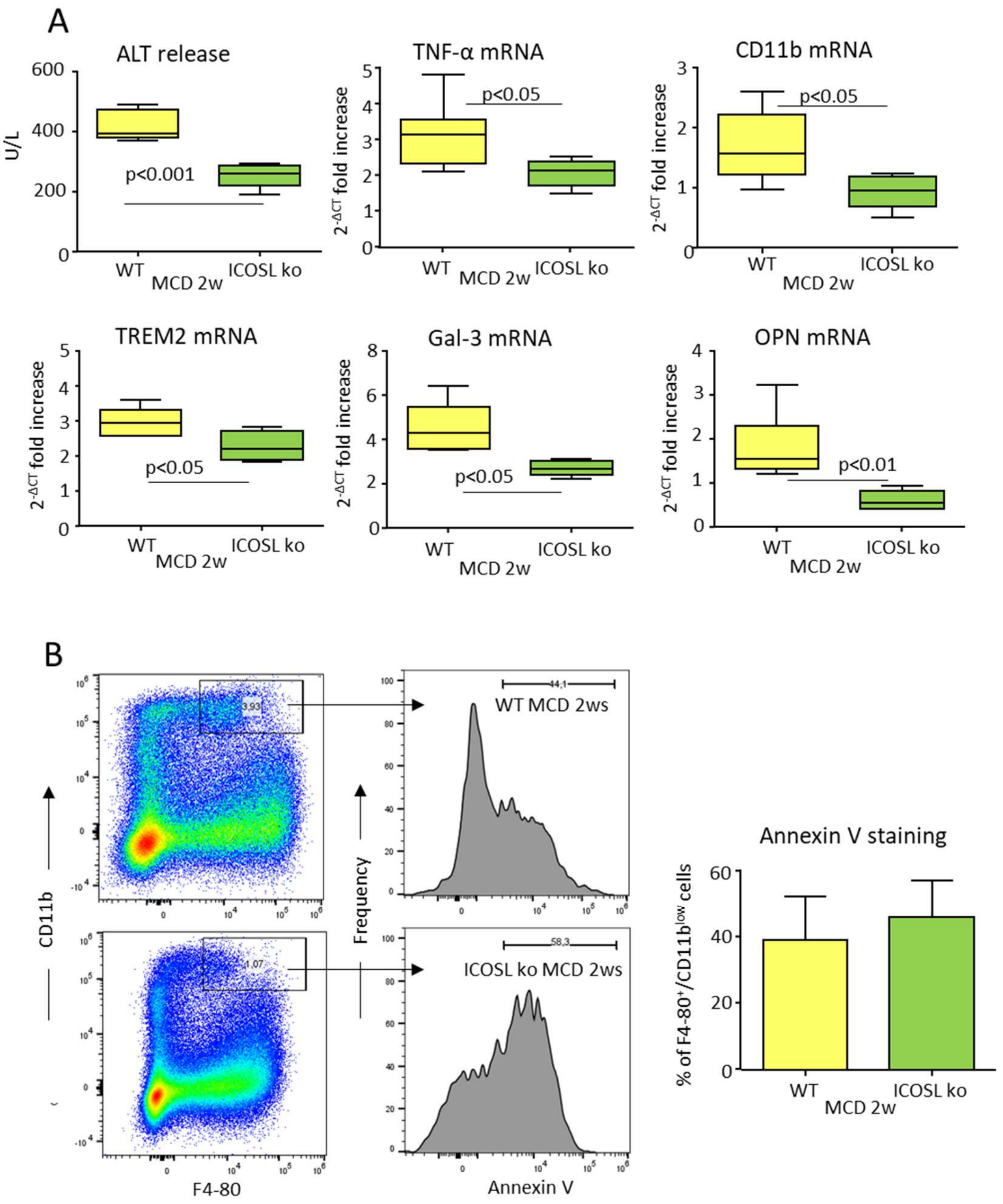

Supplementary Figure 5: Effects of ICOSL ablation on the onset of experimental MASH in mice

Wild type C57BL/6 mice received a methionine/choline deficient (MCD) or control diets for 2 weeks. (Panel A) Changes in alanine aminotransferase (ALT) release and the hepatic mRNA levels of TNF-α, CD11b, TREM2, galectin-3 (Gal-3) and osteopontin (OPN). RT-PCR values are expressed as fold increase of $2^{-\Delta CT}$ over the relative control samples. The values refer to 4-5 animals per group and the boxes include the values within 25th and 75th percentile, while the horizontal bars represent the median. The extremities of the vertical bars (10th-90th percentile) include 80% of the values. (Panel B) Flow cytometry analysis of the expression of the apoptosis marker annexin V by CD11b^{high}/F4-80^{+} monocyte/macrophages (MoMFs). The values refer to 4 animals per group ±SD.
